# Comparison of Newtonian and Non-newtonian Fluid Models in Blood Flow Simulation in Patients With Intracranial Arterial Stenosis

**DOI:** 10.3389/fphys.2021.718540

**Published:** 2021-09-06

**Authors:** Haipeng Liu, Linfang Lan, Jill Abrigo, Hing Lung Ip, Yannie Soo, Dingchang Zheng, Ka Sing Wong, Defeng Wang, Lin Shi, Thomas W. Leung, Xinyi Leng

**Affiliations:** ^1^Department of Medicine and Therapeutics, The Chinese University of Hong Kong, Hong Kong, China; ^2^Department of Imaging and Interventional Radiology, The Chinese University of Hong Kong, Hong Kong, China; ^3^Research Centre for Intelligent Healthcare, Coventry University, Coventry, United Kingdom; ^4^Shenzhen Research Institute, The Chinese University of Hong Kong, Shenzhen, China

**Keywords:** non-Newtonian fluid, intracranial atherosclerotic stenosis, computational fluid dynamics, translesional pressure ratio, wall shear stress

## Abstract

**Background:**

Newtonian fluid model has been commonly applied in simulating cerebral blood flow in intracranial atherosclerotic stenosis (ICAS) cases using computational fluid dynamics (CFD) modeling, while blood is a shear-thinning non-Newtonian fluid. We aimed to investigate the differences of cerebral hemodynamic metrics quantified in CFD models built with Newtonian and non-Newtonian fluid assumptions, in patients with ICAS.

**Methods:**

We built a virtual artery model with an eccentric 75% stenosis and performed static CFD simulation. We also constructed CFD models in three patients with ICAS of different severities in the luminal stenosis. We performed static simulations on these models with Newtonian and two non-Newtonian (Casson and Carreau-Yasuda) fluid models. We also performed transient simulations on another patient-specific model. We measured translesional pressure ratio (PR) and wall shear stress (WSS) values in all CFD models, to reflect the changes in pressure and WSS across a stenotic lesion. In all the simulations, we compared the PR and WSS values in CFD models derived with Newtonian, Casson, and Carreau-Yasuda fluid assumptions.

**Results:**

In all the static and transient simulations, the Newtonian/non-Newtonian difference on PR value was negligible. As to WSS, in static models (virtual and patient-specific), the rheological difference was not obvious in areas with high WSS, but observable in low WSS areas. In the transient model, the rheological difference of WSS areas with low WSS was enhanced, especially during diastolic period.

**Conclusion:**

Newtonian fluid model could be applicable for PR calculation, but caution needs to be taken when using the Newtonian assumption in simulating WSS especially in severe ICAS cases.

## Introduction

Intracranial atherosclerotic stenosis (ICAS) is a major cause for ischemic stroke and transient ischemic attack (TIA) in Asian populations (Wong, 2006). In recent years, computational fluid dynamics (CFD) modeling based on conventional neurovascular imaging has been applied to simulate *in vivo* cerebral blood flow and quantify cerebral hemodynamic metrics in the presence of ICAS, which cannot be achieved with conventional neurovascular imaging alone (Liebeskind et al., 2016; Linfang Lan, 2017; Liu et al., 2018; Chen et al., 2020).

Computational fluid dynamics modeling studies have indicated that global and focal cerebral hemodynamics may play an important role in governing the risk of stroke recurrence in patients with symptomatic ICAS (Leng et al., 2014, 2019). For instance, translesional pressure ratio (PR), calculated as the ratio of the pressures distal and proximal to an ICAS lesion obtained in a CFD model, has been put forward to reflect the hemodynamic significance of ICAS (Liebeskind and Feldmann, 2013). On the other hand, the relative change of wall shear stress (WSS) at the stenotic throat as compared to WSS at proximal “normal” vessel segment, has also been proposed to reflect the hemodynamic impact of an ICAS lesion on plaque growth and rupture (Lan et al., 2020). Both indices have been associated with the risk of stroke relapse in patients with symptomatic ICAS: those with a lower PR (i.e., larger translesional pressure gradient) and excessively elevated focal WSS at the ICAS lesion had significantly higher risk of recurrent stroke despite optimal medical treatment (Leng et al., 2019).

In most of the previous CFD studies on ICAS, blood was simulated as a Newtonian fluid for simplicity (Leng et al., 2014, 2019; Nam et al., 2016; Liu et al., 2018; Chen et al., 2020), despite the fact that blood is a non-Newtonian fluid with a shear-thinning nature (Nader et al., 2019). With increasing flow velocity and shear strain rate, blood flows more smoothly (Moon et al., 2014) and its viscosity decreases toward a constant, which has been commonly used as the viscosity of blood in a Newtonian model (Jahangiri et al., 2017). However, in the low-velocity areas, the true viscosity is much higher than this constant, when non-Newtonian rheological models could simulate the blood viscosity variations in different shear strain rates (Gijsen et al., 1999; Jahangiri et al., 2017). Previous studies simulating blood flow in intracranial aneurysms, in normal aorta, and in virtual arterial stenosis models have indicated differences in the estimations of pressure and WSS based on Newtonian and non-Newtonian models (Hippelheuser et al., 2014; Rabby et al., 2014).

In this study, we therefore aimed to investigate the differences, if any, of CFD simulation results in pressure (e.g., PR) and WSS between Newtonian and non-Newtonian fluid models, in a virtual arterial stenosis model and patient-specific ICAS models; we performed static simulations on the virtual model, and both static and transient simulations on patients-specific models.

## Materials and Methods

This was a substudy of the SOpHIA study (Stroke Risk and Hemodynamics in Intracranial Atherosclerotic Disease), a cohort study conducted at three teaching hospitals to investigate cerebral hemodynamics in patients with symptomatic ICAS, using routine CT angiography (CTA)-based CFD models (Leng et al., 2019). The study was approved by local institutional review board and all patients provided informed consent. We performed static CFD simulations, separately with Newtonian and non-Newtonian (Casson and Carreau-Yasuda) fluid models, in a virtual arterial stenosis model and three patient-specific ICAS models constructed based on clinically routine CTA images. We also performed transient CFD simulations in another patient-specific ICAS model, with Newtonian and non-Newtonian fluid models. We compared hemodynamic metrics (pressure and WSS) obtained by Newtonian and non-Newtonian fluid models in each case.

### Rheological Assumptions

**FIGURE 1:**
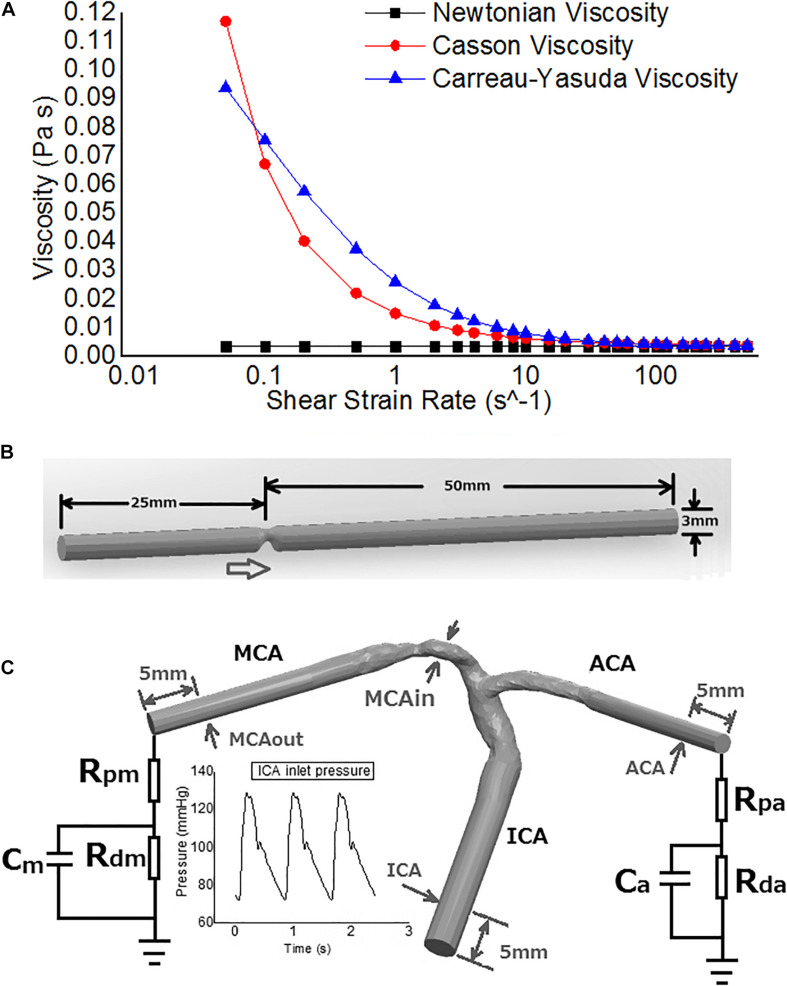
The rheological and geometrical models. **(A)** Different rheologic models. Viscosity values are derived in varied shear strain rates. The shear strain rate axis is logarithmic. **(B)** Geometry of the virtual arterial stenosis model with an eccentric 75% stenosis in area (upper). **(C)** The transient MCA stenosis (about 55% in diameter and 74% in area at the throat) model with internal carotid artery (ICA)-middle cerebral artery (MCA)-anterior cerebral artery (ACA) branches (lower). Boundary conditions: pressure on ICA inlet, Windkessel models on ACA and MCA outlets. The arrows point to the positions of pressure measurement.

The viscosity of blood in the Newtonian model was a constant: *η* = 0.0035*Pa* ⋅ *s* (Bernabeu et al., 2013). The Casson and Carreau-Yasuda models are two common non-Newtonian blood models. As a function of shear strain rate $\dot{\gamma }$, the blood viscosity η in Casson model can be expressed as that in Eq. 1 (Morales et al., 2013) and Carreau-Yasuda model in Eq. 2 (Bernabeu et al., 2013). The difference in blood viscosity among the three assumptions was more significant with lower shear strain rate (Figure 1A).


(1) $$\eta \,\left(\dot{\gamma }\right)={\left(\sqrt{\eta_{c}}+\sqrt{\tau_{c}/\dot{\gamma }}\right)}^{2}$$

where *η_c_* = 0.0035*Pa* ⋅ *s*,*τ_c_* = 0.004*Pa*.


(2) $$\eta \,\left(\dot{\gamma }\right)=\eta_{\infty }+\left(\eta_{0}-\eta_{\infty }\right)\,{\left(1+{\left(\lambda \,\dot{\gamma }\right)}^{a}\right)}^{\left(n-1\right)/a}$$

where *η*_0_ = 0.16*Pa* ⋅ *s*, *η*_∞_ = 0.0035*Pa* ⋅ *s*, *λ* = 8.2*s*, *a* = 0.64, and *n* = 0.2128.

### Geometry Reconstruction

#### Virtual Arterial Stenosis Model

Intracranial arteries are tortuous and the geometry varies between individuals. To investigate the differences in cerebral blood flow solely due to Newtonian or non-Newtonian fluid assumptions (without considering the effects of individualized arterial geometry) in CFD simulations, we first performed static simulations in a cylinder tube with 75% area stenosis (25% area remained at the stenotic throat), eccentric from the axis (Figure 1B). The radius was 1.5 mm, similar with middle cerebral artery (MCA) *in vivo*. The model was built in Solidworks 2020 (SolidWorks Co., Concord, MA, United States).

#### Static MCA Stenosis Models

Four patients with stenosed MCA recruited in the SOpHIA study (Leng et al., 2019) were analyzed in the current study. We performed static cerebral blood flow simulation in three cases and transient simulation in the remaining case.

In the SOpHIA study, cerebral CTA was performed in all patients at baseline after an acute ischemic stroke or TIA, with a 64-slice CT scanner (Lightspeed VCT, GE Healthcare) with the following protocol: intravenous contrast (Omnipaque 300) was injected via the antecubital vein at a rate of 3–3.5 mL/s with a total volume of 70 mL, and images were obtained with 120 kVp, 550 mAs, 0.625 mm slice thickness and 0.4 s rotation (Lan et al., 2020).

The 3-dimensional vessel geometry of distal internal carotid artery (ICA) bifurcation with proximal MCA and anterior cerebral artery (ACA) was reconstructed based on the CTA source images, using MIMICS 18.0 (Materialise NV, Belgium). The geometry was then smoothed with errors (self-intersections, spikes, small holes, etc.) amended in Geomagic Studio 12.0 (3D Systems, Rock Hill, SC, United States). A neurologist (Dr. Lan) compared the reconstructed 3D geometry and the CTA images to confirm the correctness of the reconstructed 3D vessel geometry. These vessel geometries were patient-specific, which possessed different tortuosity and degrees of luminal stenosis.

#### Transient MCA Stenosis Model

We simulated transient blood flow in another patient-specific model with MCA stenosis (55% diameter stenosis and 74% area stenosis; Figure 1C). The geometry of ICA-MCA-ACA bifurcation was extracted from CTA source images similarly as the patient-specific models for static simulation. To eliminate spatial fluctuations of hemodynamic parameters adjacent to the stenotic lesion, we elongated the inlet and outlets to cylinders with identical cross-section areas in Solidworks software. The smoothing was performed in Geomagic Studio software, as for patient-specific static models.

### Mesh Generation

The geometric models were input into the ANSYS software package 2019 R1 (ANSYS, Inc., Canonsburg, PA, United States) on a DELL Precision T7610 Workstation for meshing and CFD simulation. Tetrahedral elements were used for meshing.

Currently, there is no standard on the maximal element size (maximal length of the edges of a tetrahedral element in the mesh) in meshing of the intracranial artery wall, which was set as 0.25 mm in some previous studies (Ren et al., 2016; Vali et al., 2017; Lan et al., 2020). To preclude the possible effects of maximal element size on the simulation results, we conducted mesh independence study before determining the maximal element size to be adopted in the current study. On the three patient-specific models for static simulation, the pressure and WSS calculated using the meshes with the maximal element size of 0.5, 0.35, and 0.25 mm on intracranial artery wall were compared with the values derived from the mesh with maximal element size of 0.2 mm. The relative differences of area-averaged pressure and WSS were, respectively less than 1 and 3% in the simulations with maximal element sizes of 0.25 mm in each of the three cases. We therefore set the maximum element size as 0.25 mm globally and 0.1 mm at inlet and outlets as in the SOpHIA study (Leng et al., 2019; Lan et al., 2020). In the idealized virtual model, there were 372,567 nodes and 1,899,831 tetrahedral elements in the mesh. The number of element was larger than 410,000 in the meshes of all the three patient-specific models for static simulation. In the transient model, there were 608,462 nodes and 3,409,255 elements.

### Boundary Conditions and Computational Simulation

The meshes were input in ANSYS CFX software for CFD simulation and post-processing. With the arterial diameter of 3 mm and flow velocity of 140 cm/s at the stenotic throat, the Reynolds number is $R\,e=\frac{\rho \,V\,D}{\mu }\approx 1484$, within the range of laminar flow (Re < 2000). Therefore, the Navier–Stokes equations were solved using finite volume method with the following settings and assumptions: (1) The fluid domain was simulated with incompressible, steady, and laminar flow assumption; (2) The density of blood was 1060 kg/m^3^; (3) The solid wall assumption was adopted on the artery wall; (4) The convergence criteria was 1.0e-4. The boundary conditions were set separately in different models.

On the virtual model, we simulated the average blood flow in a cardiac cycle. The inlet pressure was set as 110 mmHg while the mean velocity at the outlet was set as 35 cm/s (Liu et al., 2018). For each state, we performed the simulations with Newtonian, Casson and Carreau-Yasuda models.

In the patient-specific models for static simulation, the inlet pressure was set as 110 mmHg. Based on modified *in vivo* measurements, the mean velocities at MCA and ACA outlets were set as 35 and 31 cm/s, respectively to estimate the mass flow rates, which were in accordance with the Murray’s law (Moore et al., 2006; Liu et al., 2018).

In the patient-specific model for transient simulation, we imposed physiological pressure waveform (range: 72–129 mmHg, Figure 1C) at the ICA inlet (Sarrami-Foroushani et al., 2015). Due to the lack of *in vivo* measurements of flow velocity at MCA/ACA outlets, we adopted 3-element Windkessel models, to avoid possible errors caused by possibly inaccurate assumptions in blood pressure or flow rate (Figure 1C). Parameters of the Windkessel models were based on physiological measurements that had been used in previous studies on cerebral arteries (Alastruey et al., 2007). Three transient simulations were conducted over three cardiac cycles (with a time step length of 0.005 s) with Newtonian, Casson, and Carreau-Yasuda rheological assumptions separately.

### Measurement of Hemodynamic Metrics

Translesional PR was calculated as the ratio of post-stenotic pressure and pre-stenotic pressure (Feng et al., 2020). In the virtual model, to avoid any effect of unstable flow around the stenosis, the locations of pressure measurement were 5 mm from the inlet and the outlet. In the patient-specific models, the measurement were performed at the arterial segments away from the stenotic lesion where blood flow was possibly stable.

Wall shear stress is highly dependent on the local flow field. Considering the effect of arterial geometry (tortuosity, change of diameter, etc.) on the local flow field, WSS values were only measured in patient-specific models.

We compared the translesional PR values and WSS measures obtained with the Newtonian and non-Newtonian models, and calculated the relative between-model difference in each metric: (value from Newtonian model–value from non-Newtonian model)/value from non-Newtonian model. For each metric of interest, we also calculated the ratio of the area with high relative difference between the models (using >10% and >20% as the threshold for dichotomization) and the area of the entire model. To investigate the cylic changes of WSS, we chose two points from the areas of high and low WSS values, respectively, and observed the waveform of WSS in a cardiac cycle.

## Results

### Virtual Model of Arterial Stenosis: Static Simulation

The PR values derived by Newtonian, Casson, and Carreau-Yasuda assumptions, were 0.914, 0.913, and 0.914. Between three rheological assumptions, the differences of PR value were within 1%. The difference in PR caused by different rheological assumptions was negligible in this virtual model.

### Patient-Specific ICAS Models: Static Simulation

**FIGURE 2:**
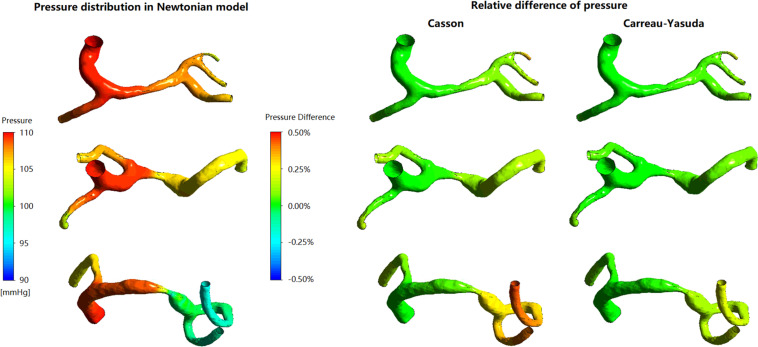
The distribution of pressure in Newtonian model, and corresponding relative differences distribution in non-Newtonian models. The relative difference delineates the degrees of deviation of Newtonian results from the non-Newtonian results.

In the three patient-specific models of ICAS cases, the relative difference in pressure was less than 1% throughout the arterial wall (Figure 2). The difference between Newtonian and non-Newtonian rheological assumptions in pressure distribution (therefore PR) was negligible.

**FIGURE 3:**
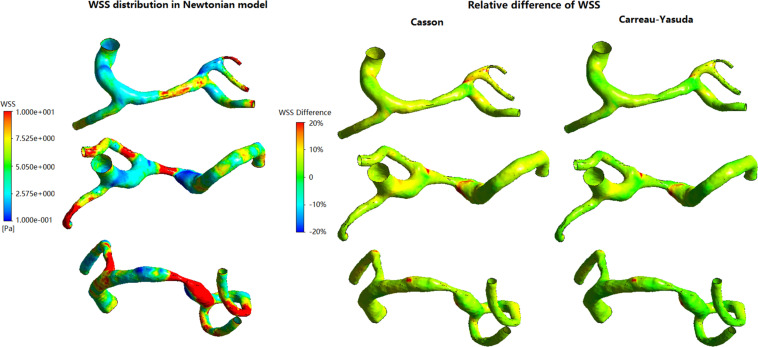
The distribution of WSS in Newtonian model, and corresponding relative difference distributions in non-Newtonian models.

In Figure 3, in some areas with low WSS, large differences (>10%) between Newtonian and non-Newtonian assumptions in WSS were observed in some low-WSS areas. However, in all the three cases, the areas with the difference in WSS between Newtonian and non-Newtonian assumptions larger than 10 and 20%, were less than 7 and 1.5% of the whole surface, respectively (Table 1). Therefore, the difference between Newtonian and non-Newtonian rheological assumptions in WSS distribution was limited in static simulations on patient-specific models with ICAS.

**TABLE 1:** The areas (in percentage) in each case with the relative difference between Newtonian and non-Newtonian models larger than 10 and 20%.

Cases	Stenosis ratio in area	Area (in percentage) with WSS relative difference >10%		Area (in percentage) with WSS relative difference >20%	
		Casson	Carreau-Yasuda	Casson	Carreau-Yasuda
Case 1	37.4%	6.88%	3.02%	1.12%	0.73%
Case 2	67.1%	5.77%	2.37%	0.78%	0.47%
Case 3	84.2%	5.47%	2.05%	0.67%	0.22%

### Patient-Specific ICAS Model: Transient Simulation

#### Pressure and PR Values

**FIGURE 4:**
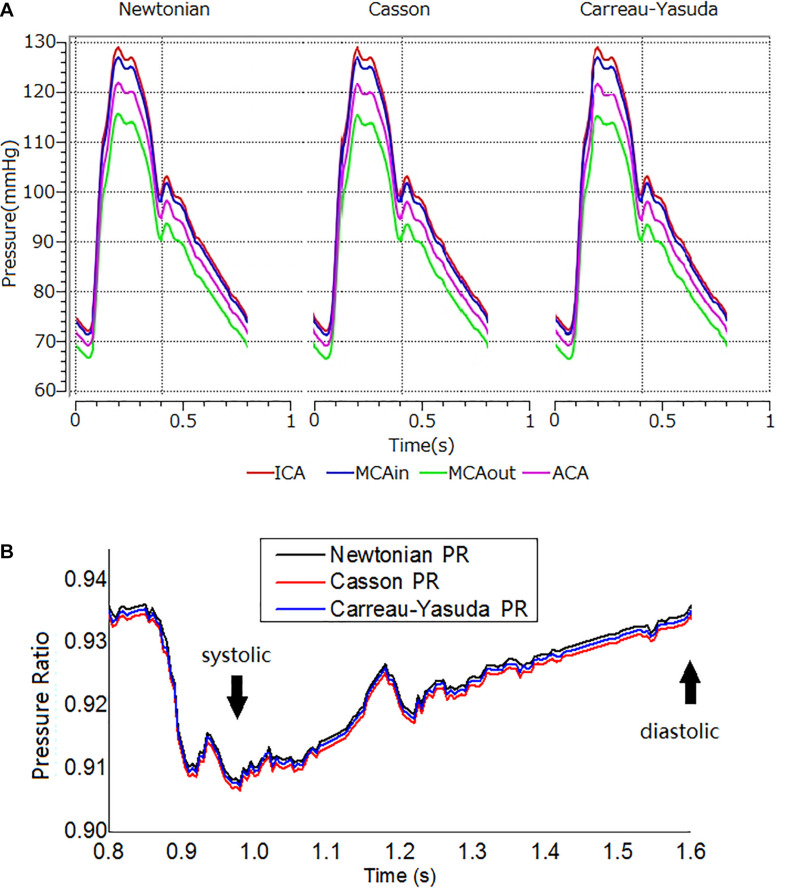
The transient pressure and PR distribution. **(A)** Transient pressure curves in the ICA-MCA-ACA branching model with Newtonian, and Casson, and Carreau-Yasuda rheological models in a cardiac cycle. The simulations lasted for three cardiac cycles. The results are from the second cardiac cycle. The positions of measurement are shown in Figure 1C. **(B)** The transient PR curves in Newtonian, Casson, and Carreau-Yasuda models during the second cardiac cycle. PR was calculated as the area-averaged pressure at MCAout divided by the area-averaged pressure at MCAin (locations of MCAout and MCAin are shown in Figure 1C).

Figure 4A showed transient pressure curves during a cardiac cycle at ICA inlet, MCA inlet (MCAin), MCA outlet (MCAout), and ACA outlet. The pressure of MCAout was the lowest due to the translesional pressure drop. There is no observable difference between the pressure curves of Newtonian and non-Newtonian assumptions.

In diastole, the maximum relative difference in pressure on the artery wall between Newtonian and non-Newtonian assumptions on the vessel wall were 0.26 and 0.14% for Casson and Carreau-Yasuda assumptions, respectively. In systole, the corresponding values were 0.20 and 0.09% for Casson and Carreau-Yasuda assumptions.

Newtonian, Casson, and Carreau-Yasuda PR curves were comparable in systole, with minor differences in late diastole (Figure 4B). The difference between Newtonian and non-Newtonian rheological assumptions in PR value was negligible in the transient simulation on the patient-specific model with ICAS.

#### WSS Distribution

**FIGURE 5:**
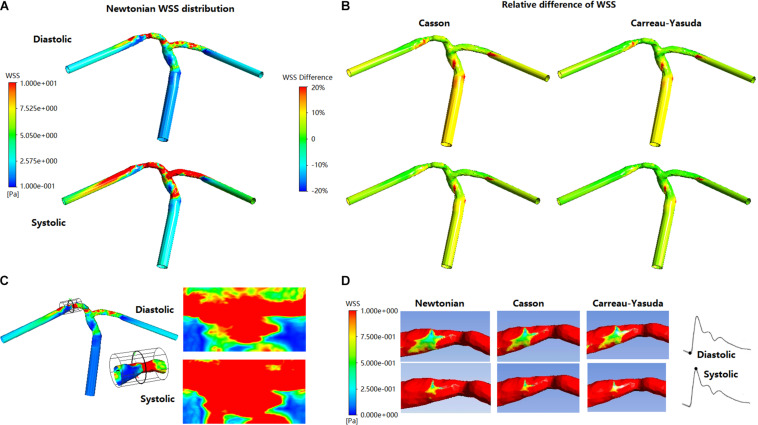
The distribution of WSS at end of diastole and systolic peak in Newtonian, Casson, and Carreau-Yasuda models. **(A)** WSS distribution in Newtonian model. **(B)** The relative difference between non-Newtonian (Casson/Carreau-Yasuda) and Newtonian models. **(C)** The expansion (cylindrical projection) of stenosis WSS distribution in Newtonian model. The scale is identical with panel **(A)**. **(D)** The stenotic region was enlarged in the low panel to reveal the low-WSS areas (red areas were those with WSS > 1 Pa).

In all simulations the highest WSS areas existed at the throat of MCA stenosis (Figure 5A). WSS distribution fluctuated obviously during a cardiac cycle (Figures 5B,C). Between Newtonian and non-Newtonian assumptions, large differences (higher than 20%) in WSS appeared in less than 6% area of vessel wall in systole, but quadrupled in diastole (Figures 5B,D). In diastole, compared with Newtonian results, the percentage of vessel wall area with difference in WSS higher than 10 and 20% were 37.56 and 1.32% for Casson assumption, while 8.29 and 0.69% for Carreau-Yasuda assumption. In systole, the corresponding results were 5.40 and 1.09% for Casson assumption, while 2.03 and 0.59% for Carreau-Yasuda assumption. Higher differences in WSS appeared in the areas with low WSS values.

The rheological influence on minimum WSS was observable (Table 2). Between Newtonian and non-Newtonian models, the difference in maximum WSS was within 8%, while the difference in minimum WSS exceeded 40%. The difference between Newtonian and non-Newtonian rheological assumptions was obvious in low-WSS (lower than 0.1 Pa) areas (Figure 5D).

**TABLE 2:** Maximum and minimum WSS values in a MCA stenosis model with Newtonian and non-Newtonian assumptions in transient CFD simulation.

	Systolic			Diastolic		
	Newtonian	Casson	Carreau-Yasuda	Newtonian	Casson	Carreau-Yasuda
Max WSS	6.368	6.836 (7.3%)	6.718 (5.4%)	1.31	1.335 (1.9%)	1.318 (0.6%)
Min WSS	1.263e-2	1.698e-2 (34.4%)	9.416e-3 (25.1%)	2.279e-2	6.7e-3 (70.6%)	3.29e-2 (44.3%)

*In non-Newtonian results, the relative differences compared with Newtonian model were in brackets.*

**FIGURE 6:**
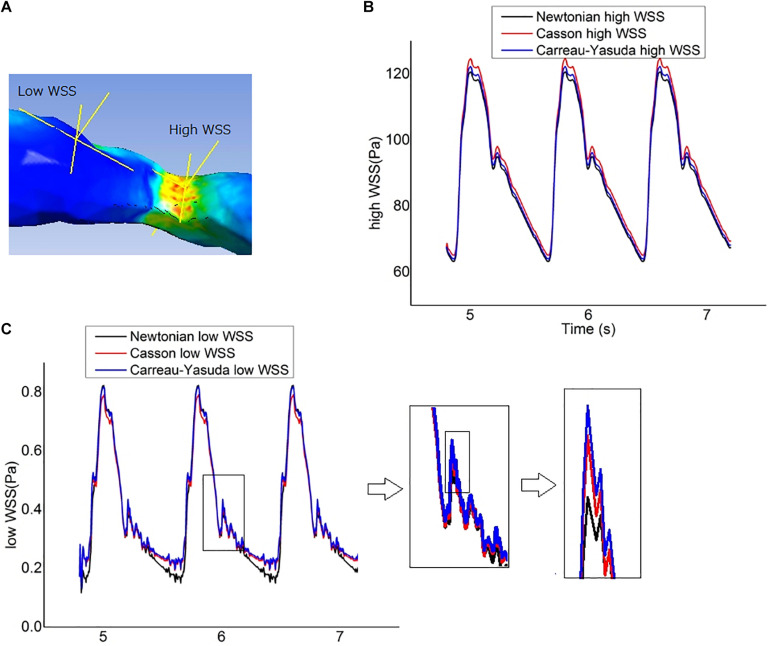
Wall shear stress (WSS) measurement. **(A)** The locations of two points chosen to represent the high-WSS and low-WSS areas. **(B)** Curves of transient WSS during three cardiac cycles in Newtonian and non-Newtonian models, as measured at the representative high-WSS location. **(C)** Curves of transient WSS during three cardiac cycles as measured at the representative low-WSS location, which diverged in late diastole in Newtonian and non-Newtonian models.

The fluctuations of the WSS in a cardiac cycle was observed at two points from the areas with high and low WSS (Figure 6A). There was no significant between-model difference in the WSS waveforms for the high-WSS point (Figure 6B). For the low-WSS point, the differences between Newtonian and non-Newtonian models were more significant in late diastole, with a relative difference larger than 10%, where the WSS fluctuated between consecutive time steps (Figure 6C).

## Discussion

In this study, using virtual and patient-specific models, we investigated the effects of Newtonian and non-Newtonian (Casson and Carreau-Yasuda) fluid assumptions on computational simulation results of cerebral hemodynamics in the presence of ICAS. We found no significant difference in pressure distribution or translesional PR obtained by the different assumptions in virtual or static/transient patient-specific models. The difference in WSS distribution was limited in static patient-specific ICAS models, which, however, was considerable in the low-WSS regions of the transient patient-specific models especially during late diastole.

Despite the shear-thinning effect of blood, currently there is no consensus on non-Newtonian fluid model for blood flow simulation (Hippelheuser et al., 2014). There are many non-Newtonian blood models such as Casson, Carreau-Yasuda, Cross, Power-law, and Quemada models (Sochi, 2013). The Quemada model is mainly used for the simulation of microcirculation in arterioles and capillaries, while the Cross model could derive far different results with other non-Newtonian models (Karimi et al., 2014; Sriram et al., 2014). In contrast, the Casson and Carreau-Yasuda models have been widely applied in the simulation of arterial blood flow (Bernabeu et al., 2013; Morales et al., 2013), which therefore were used in this study. We did not observe any significant difference between Newtonian and non-Newtonian assumptions in estimating translesional pressure drops or PR values (representing the relative translesional pressure drop). Our results were in accordance with theoretical analysis and previous CFD studies. Theoretically, the translesional pressure drop (ΔP) caused by the stenosis can be expressed as a quadratic function of flow rate (Q): ΔP = *AQ*^2^ + *BQ*, where A and B are parameters associated with the stenosis geometry and blood viscosity. The quadratic item shows the effects of turbulence on energy dissipation caused by stenosis (Young and Tsai, 1973). At the stenotic throat where the velocity and shear strain rate are high, the Newtonian and non-Newtonian assumptions have similar viscosity values (Figure 1A). Thus, the effect of different rheological assumptions on PR estimation is limited. Some previous CFD studies on the blood flow of other arteries had also concluded that Newtonian and non-Newtonian assumptions can derive similar results in pressure distributions (Amornsamankul et al., 2006; Mamun et al., 2016). However, the high tortuosity and varying diameter of intracranial arteries may influence the pressure drop. To our best knowledge, this study was among the first to investigate the rheological effect of Newtonian and non-Newtonian assumptions on pressure and PR estimations in the presence of ICAS.

Wall shear stress is an important hemodynamic factor in the development and progression of ICAS. Low and oscillatory WSS is related with initiation and early development of atherosclerosis, while high WSS upon an atherosclerotic plaque might increase the risk of plaque instability (Peiffer et al., 2013). Previous studies had conflicting results on the effects of Newtonian and non-Newtonian assumptions on WSS distributions in simulating arterial blood flow; some found similar between-model results (Johnston et al., 2006; Bernabeu et al., 2013), while others studies derived opposite conclusions (Karimi et al., 2014; Sriram et al., 2014). Furthermore, the geometry of intracranial arteries especially in ICAS cases would also have complex influence on the distribution of WSS in different rheological assumptions. Therefore, we investigated the effects of Newtonian and non-Newtonian assumptions on WSS distributions in ICAS cases in this study.

The Newtonian and non-Newtonian rheological assumption had been compared in the CFD simulation of intracranial and extracranial (e.g., carotid) arteries (Xiang et al., 2012; Gambaruto et al., 2013; Morales et al., 2013; Frolov et al., 2016; Guerciotti and Vergara, 2018; Valen-Sendstad et al., 2018; Saqr et al., 2019). Morales et al. (2013) simulated the area-averaged WSS on three patient-specific models with intracranial aneurysms in a steady state based on a time-averaged inlet flow rate, and found that the maximal difference in area-averaged WSS between non-Newtonian (Casson) and Newtonian rheological assumptions was 12%. Frolov et al. (2016) simulated the WSS on intracranial aneurysm at the end of systole, where the WSS varied from 3.52 mPa to 10.21 Pa for the Newtonian rheological model, and 2.94 mPa to 9.14 Pa for the non-Newtonian model. As a result, the relative difference in minimum WSS (16.4%) was much higher than that in maximal WSS (10.5%). Xiang et al. (2012) reported that the Casson and Newtonian rheological assumptions derived similar time-averaged WSS on most areas of an intracranial aneurysm, but the difference reached 55% on dome area where the WSS was low. In carotid artery studies, Guerciotti and Vergara (2018) found that the difference in area-averaged WSS between non-Newtonian (Carreau-Yasuda) and Newtonian rheological assumptions was within 10% during systole but increased to 18.4% during diastole. These previous findings in general agreed with our findings. In our results, the difference between Newtonian and non-Newtonian assumptions in WSS was not significant in static simulations. In the transient simulation, the WSS values derived by the three rheological assumptions were also comparable in high-WSS areas, whereas the differences were noteworthy in low-WSS areas, especially during late diastole when WSS was low (Figure 6). Moreover, the difference in WSS between Carreau-Yasuda and Newtonian assumptions was smaller than that between Casson and Newtonian assumptions, which was in accordance with the rheological properties of the three assumptions as shown in Figure 1A.

According to this and previous relevant studies, the Newtonian assumption would be applicable for WSS estimation in normal intracranial arteries as long as the Reynolds number is within the range of laminar flow (Re < 2000), which appears in most intracranial arteries with ICAS (Lee and Steinman, 2007; Samady et al., 2011). However, caution needs to be taken when the Newtonian assumption is applied in some extreme cases (Re > 2000, which may appear locally due to a stenosis), particularly in estimating the WSS values in low-WSS regions. This is in accordance with existing studies on CFD simulation of arteries with stenosis or aneurysm when the abnormal geometry altered focal hemodynamics, e.g., the formation of vortices and recirculation zone, with significant rheological effect on WSS distribution (Cho and Kensey, 1991; Hippelheuser et al., 2014; Liu et al., 2017). Overall, the current study on intracranial stenosis and previous studies on intracranial aneurysms suggest that the choice of rheological assumption impacts the results in simulating cerebral hemodynamics in low-WSS areas (Gambaruto et al., 2013). To achieve reliable WSS estimation in such scenarios, non-Newtonian assumptions should be considered.

Another factor that may have impact on the simulation results is the velocity conditions used. The Womersley velocity profile has been widely applied in the CFD simulation of blood flow in proximal major arteries. However, intracranial arteries are highly curved, which significantly influences the velocity profile. It was found that the variations of Womersley number only slightly affects the normalized WSS (maximum of 14%) in simulating hemodynamics in intracranial aneurysms (Asgharzadeh and Borazjani, 2016). A recent study also found that Womersley number has minimal effect on time-averaged aneurysm circulation compared with Dean and Reynolds numbers (Barbour et al., 2021). Furthermore, it was suggested that the difference between the Poiseuille and Womersley solutions is less significant in the arteries far from the heart such as cerebral arteries, where parabolic velocity distribution is a permissible approximation (Ugron and Paál, 2014). Therefore, in the current study, we extended the inlet segment of the model to have fully developed flow in the models, rather than adopt the Womersley velocity profile in the simulation.

This study had limitations. Firstly, we adopted the solid wall assumption while *in vivo* arterial walls are elastic; but the compliance of intracranial arteries is less than that of aorta and common carotid artery by 1–2 orders of magnitude (Zhang et al., 2014), and we adopted the pressure waveform of ICA in the transient simulations in which the compliance of aorta and large arteries had been incorporated. Secondly, the rheological properties of blood vary between individuals, but in this study the boundary conditions and rheological properties of blood and the parameters in the Casson and Carreau-Yasuda assumptions were not patient-specific, due to the lack of *in vivo* measurements. Additionally, in this pilot study we used unstructured mesh, whilst enhancing the mesh in the near wall zone with boundary layers could better capture the near wall behavior of the flow and may provide more accurate estimation of the WSS measures in future studies. Finally, only three cases were analyzed in this pilot study. More cases are needed for further validation of the findings and for correlation with the clinical outcomes. In future studies, compliance of arterial walls, mesh enhancement, patients-specific boundary conditions (e.g., velocity profile derived from dynamic clinical imaging) and rheological properties, and a larger-scale validation, could be considered to achieve more reliable estimations of the cerebral hemodynamic parameters, and to reveal the differences between Newtonian and non-Newtonian assumptions in cerebral blood flow simulation results in ICAS cases.

## Conclusion

The study indicated negligible difference in pressure distribution in ICAS cases between CFD models with Newtonian and non-Newtonian fluid assumptions. Regarding the WSS simulation results, the difference between Newtonian and non-Newtonian models was trivial in high-WSS area but considerable in low-WSS area and in late diastole in a cardiac cycle. Therefore, in cerebral blood flow simulation in ICAS patients, the Newtonian fluid assumption could be applied in pressure estimation, and WSS estimation in high- or normal-WSS regions, but caution needs to be taken when using the Newtonian assumption in estimating WSS in low-WSS regions in such cases.

## Data Availability Statement

The original contributions presented in the study are included in the article/supplementary material, further inquiries can be directed to the corresponding author/s.

## Ethics Statement

The studies involving human participants were reviewed and approved by The Joint Chinese University of Hong Kong–New Territories East Cluster Clinical Research Ethics Committee. The patients/participants provided their written informed consent to participate in this study.

## Author Contributions

HL performed design the details of this study and performed the simulations, and drafted the manuscript. LL, HI, TL, and XL analyzed the clinical data and selected the cases for study. DZ and XL critically reviewed and edited the manuscript. XL and LS supervised the project that led to production of the results shown here. All authors contributed to the discussion and manuscript revision and concur with the current submitted version.

## Conflict of Interest

The authors declare that the research was conducted in the absence of any commercial or financial relationships that could be construed as a potential conflict of interest.

## Publisher’s Note

All claims expressed in this article are solely those of the authors and do not necessarily represent those of their affiliated organizations, or those of the publisher, the editors and the reviewers. Any product that may be evaluated in this article, or claim that may be made by its manufacturer, is not guaranteed or endorsed by the publisher.
