# Corrigendum: Comparison of Newtonian and Non-newtonian Fluid Models in Blood Flow Simulation in Patients With Intracranial Arterial Stenosis

**DOI:** 10.3389/fphys.2021.782647

**Published:** 2021-10-19

**Authors:** Haipeng Liu, Linfang Lan, Jill Abrigo, Hing Lung Ip, Yannie Soo, Dingchang Zheng, Ka Sing Wong, Defeng Wang, Lin Shi, Thomas W. Leung, Xinyi Leng

**Affiliations:** ^1^Department of Medicine and Therapeutics, The Chinese University of Hong Kong, Hong Kong, China; ^2^Department of Imaging and Interventional Radiology, The Chinese University of Hong Kong, Hong Kong, China; ^3^Research Centre for Intelligent Healthcare, Coventry University, Coventry, United Kingdom; ^4^Shenzhen Research Institute, The Chinese University of Hong Kong, Shenzhen, China

**Keywords:** non-Newtonian fluid, intracranial atherosclerotic stenosis, computational fluid dynamics, translesional pressure ratio, wall shear stress

In the published article, there was an error regarding the affiliations for Dr. Xinyi Leng. As well as having affiliation 1, Dr. Leng should also have “4. Shenzhen Research Institute, The Chinese University of Hong Kong, Shenzhen, China”.

The authors apologize for this error and state that this does not change the scientific conclusions of the article in any way. The original article has been updated.

## Publisher's Note

All claims expressed in this article are solely those of the authors and do not necessarily represent those of their affiliated organizations, or those of the publisher, the editors and the reviewers. Any product that may be evaluated in this article, or claim that may be made by its manufacturer, is not guaranteed or endorsed by the publisher.

